# Enzyme-Responsive Nanoparticles and Coatings Made from Alginate/Peptide Ciprofloxacin Conjugates as Drug Release System

**DOI:** 10.3390/antibiotics10060653

**Published:** 2021-05-29

**Authors:** Yannick Bourgat, Carina Mikolai, Meike Stiesch, Philipp Klahn, Henning Menzel

**Affiliations:** 1 Institute for Technical Chemistry, Technische Universität Braunschweig, Hagenring 30, 38106 Braunschweig, Germany; y.bourgat@tu-braunschweig.de; 2 Department of Prosthetic Dentistry and Biomedical Material Science, Hannover Medical School, Carl-Neuberg-Straße 1, 30625 Hanover, Germany; mikolai.carina@mh-hannover.de (C.M.); stiesch.meike@mh-hannover.de (M.S.); 3 Lower Saxony Centre for Biomedical Engineering, Implant Research and Development (NIFE), Stadtfelddamm 34, 30625 Hanover, Germany; 4 Institute of Organic Chemistry, Technische Universität Braunschweig, Hagenring 30, 38106 Braunschweig, Germany; p.klahn@tu-braunschweig.de

**Keywords:** enzyme triggered release, drug release system, ciprofloxacin, nanoparticle, implant coating, peri-implantitis, copper-free click chemistry

## Abstract

Infection-controlled release of antibacterial agents is of great importance, particularly for the control of peri-implant infections in the postoperative phase. Polymers containing antibiotics bound via enzymatically cleavable linkers could provide access to drug release systems that could accomplish this. Dispersions of nanogels were prepared by ionotropic gelation of alginate with poly-l-lysine, which was conjugated with ciprofloxacin as model drug via a copper-free 1,3-dipolar cycloaddition (click reaction). The nanogels are stable in dispersion and form films which are stable in aqueous environments. However, both the nanogels and the layers are degraded in the presence of an enzyme and the ciprofloxacin is released. The efficacy of the released drug against *Staphylococcus aureus* is negatively affected by the residues of the linker. Both the acyl modification of the amine nitrogen in ciprofloxacin and the sterically very demanding linker group with three annellated rings could be responsible for this. However the basic feasibility of the principle for enzyme-triggered release of drugs was successfully demonstrated.

## 1. Introduction

Drug delivery systems based on hydrogel micro- and nanoparticles are of particular interest because of their good biocompatibility and good compatibility even with sensitive active ingredients such as therapeutic proteins [1,2]. Such hydrogel systems can be designed to be responsive to stimuli such as pH [3], temperature [4], redox [5], electricity [6], or magnetic fields [7]. Specific enzymes can also trigger the drug release and resulting in a highly specific on-demand delivery system [8]. This kind of triggered release could be particularly beneficial in preventing implant-associated infections [9]. Unfortunately, medical implants are a significant source of nosocomial infections also during the postoperative period [10]. Implant-associated infections are typically caused by biofilm-forming microorganisms. Biofilm formation takes place in four steps: initial adhesion of bacteria on implant surface, aggregation, formation of microcolonies, biofilm maturation, and dispersion of bacterial cells [11,12,13]. In the second step, an extracellular polysaccharide substance (EPS) is excreted by the bacteria and causes irreversible attachment. The EPS gives a well-organized and structured matrix for the bacteria that offers many advantages, such as protection against antibiotics [14], disinfectants, and dynamic environments [15]. Hygienic measures are the most efficient way to prevent implant-associated infections. However, inoculation with a few microorganisms during implantation already can result in biofilm formation leading to inflammation. In addition, biofilm-associated infections can occur even years after implantation [16]. In orthopedic implant-associated infections, the most commonly detectable bacteria is *Staphylococcus aureus*, a Gram-positive cocci [17].

Most infections in humans start with an asymptomatic period, so that the infection will be detected at a late time point. Thus, it is important to find a system that is able to fight the infection before symptoms occur and a fully formed biofilm develops. Numerous strategies have been attempted to prevent implant-associated infections. Among them are implant-coating materials, which can release antibiotics in a controlled manner [18]. Such release systems can ensure a high concentration of active ingredient at the implant without the need for very high, possibly toxic concentrations of the antibiotic to be administered systemically [19]. Those systems can be based on polymeric nanoparticles generated by biocompatible materials such as chitosan, alginate, xanthan gum, cellulose, or synthetic polymer poly-ε-caprolactone, polyacrylamide, and polyacrylate [20]. Particle formation can proceed via different mechanisms [21]: desolvation, emulsion/solvent extraction, electro-spraying, and self-assembly through ionic gelation.

According to their structural organization, nanoparticles are classified as nanocapsules or nanospheres [22]. Nanocapsules are core-shell structures, similar to micelles and liposomes. In such systems, the drug is confined or contained within the core cavity surrounded by a polymer membrane. Both hydrophobic and hydrophilic substances can be entrapped with high efficiency. Nanospheres are matrices, where the encapsulated drug is physically and uniformly dispersed. Nanospheres can be hydrophobic particles [19] or hydrophilic nanogels [23]. Lipophilic drugs are encapsulated into the polymer matrix, and hydrophilic compounds are adsorbed at the particle’s surface or within the nanogels. For both systems, the release in a targeted tissue occurs through the processes of diffusion and degradation. These processes are directly related to the biodegradability and permeability of the particle matrix or the nanogel, respectively. The drugs can be released from solid nanoparticles by diffusion through water-filled pores, diffusion through matrix, or erosion of the degradable matrix [24]. The erosion can occur as surface erosion or as bulk erosion, when, e.g., water penetrates the polymeric matrix resulting in hydrolysis of the polymer. While surface erosion is ideal for many drug delivery applications, because of the typically well-defined release rates, bulk erosion is less predictable. [24] If diffusion is faster than degradation, this implies that the mechanism of release is governed by the diffusion process, often leading to an undesired burst release. The latter is in particular problematic when diffusion from pores occur. The rapid and initial burst release is the main challenge faced by those systems and is mainly due to weak interactions between the drug and the nanoparticle [25], or meshes in the nanogel network that are too large to retain the drug efficiently [1].

Degradation and drug release can be triggered by enzymes, temperature, or pH changes, electromagnetic radiation, and variations in ionic strength or redox potential [26]. For instance, in pathological conditions (tumors or inflammation), the expression profile of specific enzymes (proteases, phospholipases, or glycosidases) is observed and used to release the drug at a specific location [27]. Drug release systems triggered by enzymes have the advantage of high selectivity [28,29,30].

Ionic gelation is a very advantageous method for the preparation of nanogel particles due to the mild conditions which allow encapsulation of sensitive therapeutic proteins [31]. Tolle et al. described nanoparticles prepared via ionic gelation of negatively charged alginate and a positively charged enzyme cleavable peptide [32]. The cleavage was triggered by a specific enzyme released during an inflammation and induced the drug release. However, the retention of the model protein interferon β was limited and a strong burst release was observed [32]. Based on these promising results and in an effort to address the main drawback of unspecific burst release, this study presents a new enzyme-responsive alginate/peptide nanogel, in which the model drug ciprofloxacin is conjugated to the peptide linker. For the preparation, ciprofloxacin was modified with a cyclooctyne moiety suitable for copper-free click reaction and conjugated with an enzyme-cleavable peptide sequence. Since this was an early stage study, poly-l-lysin (PLL) was used as a peptide sequence to identify the ability of conjugated peptide to generate nanoparticles alginate. The particle size and enzymatic degradation were studied via dynamic light scattering (DLS). Ultraviolet-visible spectroscopy (UV-Vis) was used to determine the amount of ciprofloxacin released during the degradation process. Finally, the nanoparticles were coated on titanium substrates, a material which is typically used for musculoskeletal or dental implants. Ellipsometry was used to measure the layer thickness and degradability of the coating. Attention was also placed on how conjugation and the linker fragments remaining on the drug after degradation affect the antimicrobial activity of ciprofloxacin.

## 2. Results

The aim of the investigation was to find a general synthetic route to conjugate drugs to linkers suitable for the preparation of nanogels by inotropic gelation. Alginate as polyanion and poly-l-lysine as polycation were chosen as components for the ionotropic gelation [32]. Ciprofloxacin was chosen as a model drug with broad spectrum antibacterial activity as it is readily available and it is well-known that its structure-activity relationships enable structural modification, not negatively impacting the antibacterial activity [33]. Indeed, it is known that the carboxylic acid and the carbonyl group are essential for the formation of the complex with the gyrase bound to DNA [34]. If these groups are chemically modified or removed, the antimicrobial activity is strongly reduced [35]. However, modifications at the piperazine ring via alkylation or acylation can result in highly active derivatives [33,36,37]. Therefore, the conjugation was carried out by modification of the piperazine ring.

### 2.1. Conjugation of the Ciprofloxacin with Poly-l-Lysine

**Figure 1:**
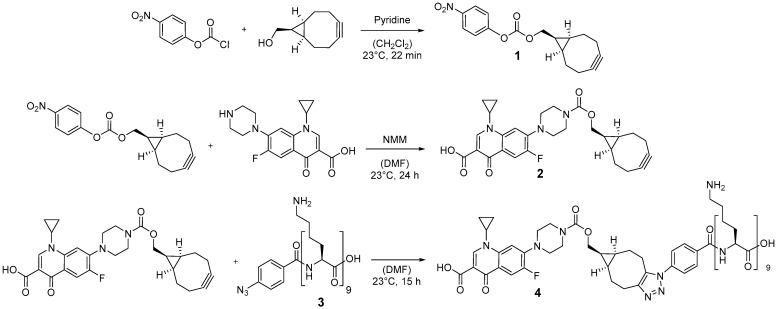
Reaction scheme for the preparation of ciprofloxacin conjugated to a poly-l-lysin sequence which can be used as linker.

In order to conjugate ciprofloxacin to the poly-l-lysine (PLL) sequence a versatile copper-free 1,3-dipolar cycloaddition (click reaction) was chosen, holding potential for binding various antimicrobial drugs. Compared to copper-catalyzed Huisgen cycloaddition, cyclooctynes are reported to react selectively with azides without any copper(I) catalyst avoiding cytotoxic effects of copper(I) in the product [38]. The synthetic route for the preparation of the ciprofloxacin to the PLL-linker is depicted in Figure 1.

First, (1R,8S,9s)-bicyclo[6.1.0]non-4-yn-9-yl-methanol (BCN-OH) was reacted with *p*-nitrophenyl chloroformate in the presence of pyridine to obtain *p*-nitrophenyl carbonate **1** with 85% yield. The ^1^H-NMR was consistent with previous work [39], and all signals were assigned to the corresponding protons (see Supplementary Figure S1). Subsequently, the BCN moiety was installed at the free amino function of ciprofloxacin by reaction with the *p*-nitrophenylcarbonate **1** in the presence of *N*-methyl morpholine (NMM). In order to avoid thermal degradation of the BCN moiety, ciprofloxacin was first dissolved at 82 °C and the solution was cooled down to 23 °C before compound **1** was added. ^1^H-NMR spectroscopy was performed to prove the reaction and the structure of compound **2** (see Figure S2). Electrospray ionization mass spectrometry (ESI-MS) was used to further confirm the synthesis. The mayor peaks were found at 508.22, 530.20, and 1037.42 m/z, which represent the following ions: [M + H]^+^ = 508.22 m/z, [M + Na]^+^ = 530.20 m/z, [2 M + Na]^+^ = 1037.42 m/z. M represents the calculated mass for C_28_H_30_FN_3_O_5_ = 507.22 g/mol.

Poly-l-lysine-N_3_ **3** was synthesized using automated solid-phase-supported peptide synthesis based on an Fmoc-protection group strategy with DIC/HOBt as coupling reagents utilizing a 2-chlorotrityl resin. The final peptide was cleaved from the resin using TFA and precipitated from the cleavage solution by addition of cold diethyl ether.

Finally, the BCN-modified ciprofloxacin derivative **2** was conjugated to poly-l-lysine-N_3_ **3** via copper-free azide-alkyne click reaction (Figure 1). ^1^H-NMR, Fourier-transform infrared (FTIR), and UV-Vis measurements were used to characterize compound **4**. ^1^H-NMR spectra (see Figure S3) revealed the presence of protons which can be assigned to ciprofloxacin and poly-l-lysine. Protons H8, H7, H6, and H10/H9 belong to ciprofloxacin and the signal at 1.87–1.25 ppm represented protons of PLL (H3/4 and H5). In addition, FTIR of PLL_9_-N_3_ (**3**) and poly-l-lysine-ciprofloxacin (**4**) was performed to prove the disappearance of the signal at 2121 cm^−3^, which belongs to the reacting azide of PLL_9_-N_3_ (**3**) (See Figure S9). Finally, UV-Vis was used to confirm the presence of ciprofloxacin. An absorption peak was observed at 278 nm, which is characteristic for ciprofloxacin.

### 2.2. Nanoparticle Formation and Characterization

Nanoparticles were formed by ionotropic gelation in an aqueous solution [32] by mixing a solution of purified alginate and PLL-ciprofloxacin **4**. Due to ionic interactions between the components, they self-assembled into nanoparticles. Various parameters such as the PLL-ciprofloxacin/alginate ratio, the pH, and the concentration influenced the particle formation. The influence of the PLL-ciprofloxacin/alginate ratio was tested by mixing a solution of **4** and alginate at different ratios. The concentration of both solutions was fixed at 1 mg/mL in deionized H_2_O. DLS-conjugated measurements were used to determine particle sizes. PLL-ciprofloxacin/alginate ratios of 1:4, 1:3, 1:2, and 1:1 were tested. In all cases, monomodal particle-size distributions were obtained.

**Figure 2:**
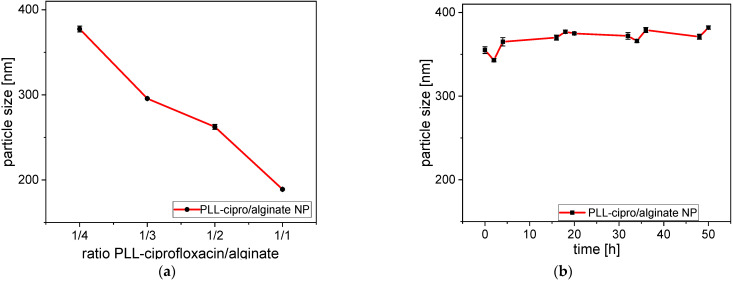
(**a**) Dependence of the size of nanogels prepared with different PLL-ciprofloxacin/alginate ratios at RT in deionized water; (**b**) size of the nanoparticles (PLL-ciprofloxacin/alginate 1/3) as a function of incubation time in phosphate buffer saline (pH 7.4) at 37 °C. The particle size for the 1/3 mixture here was somewhat higher because of the buffer and the higher temperature.

As shown in Figure 2a, the size decreased as the amount of PLL increased. Indeed, it is known that the size of the particle is influenced by different parameters such as concentration of the polycation and the polyanion [40]. Giri et al. [41] explained that ionic gelation is based on the capability of polyelectrolytes to generate crosslinks with respective counter ions to form nanoparticles. In this case, PLL acts as a cross-linker to bind the alginate chains to each other. At the same time PLL compensates the negative charges of alginate. Therefore, by increasing the amount of positive PLL, negative charges are compensated and the repulsive force inside the system is reduced. The direct consequence is a decrease of the particle size.

The polydispersity index for 1:4, 1:3, 1:2, and 1:1 mixtures were found to be 0.488, 0.225, 0.247, and 0.171, respectively. It is clear that the PDI tends to decrease with an increasing amount of polycation. This behavior has been observed by Hiorth et al. with Zn^2+^ as polycation [42]. This might be explained by the transition from free alginate to cross-linked alginate chains. In order to maximize the cost effectiveness while keeping a low polydispersity index for the NP a ratio of 1:3 was chosen to perform the stability and degradation tests. The stability was tested by incubating the nanoparticles for 50 h at 37 °C in phosphate buffered saline (Figure 2b). Due to the different conditions (pH, temperature, salt concentrations) the particle size for the 1/3 composition was somewhat higher than in the previous experiment (355 nm instead of ~300 nm). There was a slight further increase in particle size from 355 to 380 nm. However, in general the stability of the nanoparticles was confirmed.

**Figure 3:**
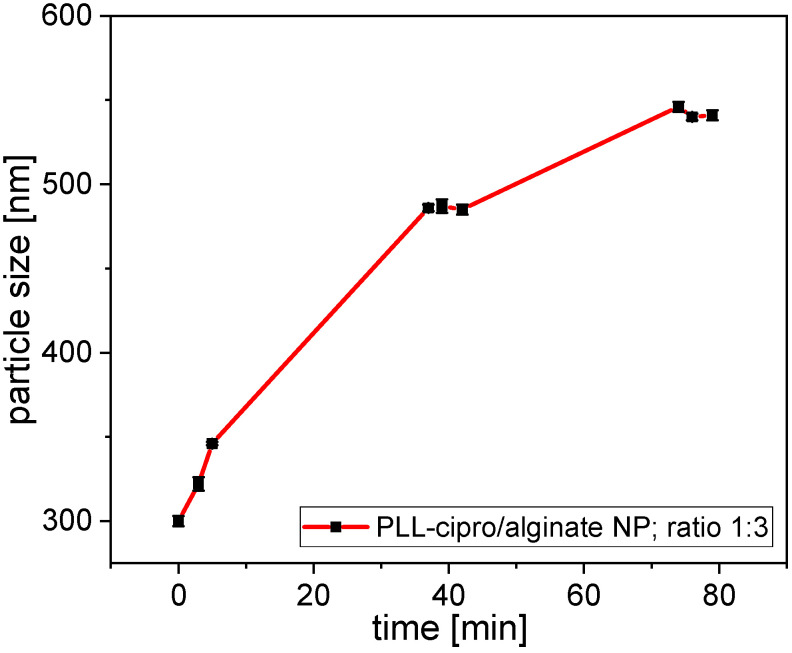
Size of the nanoparticles (PLL-ciprofloxacin/alginate 1/3) as function of incubation time in phosphate buffer saline (pH 7.4) at 37 °C after addition of trypsin (final concentrations of 2.5 µg/mL).

The degradability and release of ciprofloxacin were tested by incubating the system with trypsin (2.5 µg/mL). This enzyme is known to cleave PLL sequences [28]. The incubation led to a substantial increase in particle size (Figure 3). As was shown in Figure 2a, a decrease in the amount poly-l-lysine, thus a reduced PLL to alginate ratio led to an increase in nanoparticle size. Indeed, during incubation, trypsin converted PLL into monomer moieties, reducing the PLL concentration and as a direct consequence an increase in nanoparticle size was observed.

**Figure 4:**
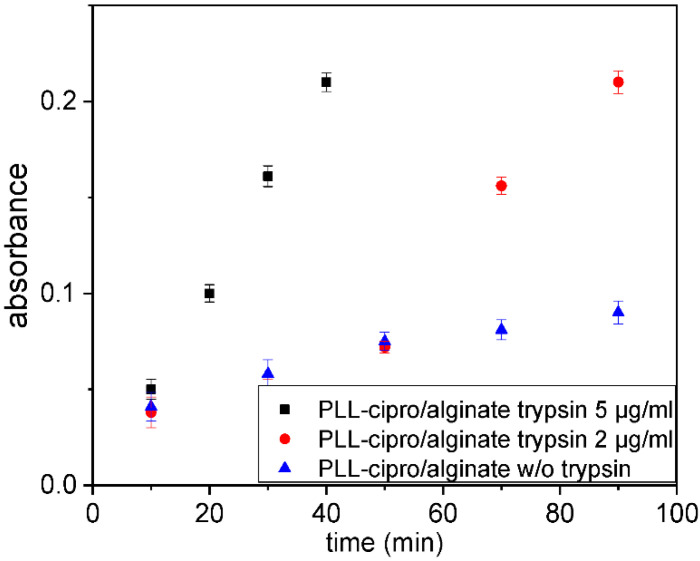
Absorbance at 278 nm, which corresponds to the ciprofloxacin concentration in the dialysate as function incubation time in PBS buffer (pH 7.4) without, with 2 µg/mL or 5 µg/mL trypsin, respectively, at 37 °C.

The triggered release of ciprofloxacin by cleavage of the peptide sequence was confirmed by the following investigations. Different dispersions of nanoparticles were incubated with trypsin in PBS at 23 °C and dialyzed to filtrate the solutions. The aim was to remove the non-degraded particles and to extract the released ciprofloxacin. The dialysate was investigated by UV-Vis to quantify the ciprofloxacin at 278 nm. To study the influence of trypsin, the experiment was tested with different concentrations (s. Figure 4). With regards to the incubation without trypsin (Figure 4, blue curve), a small amount of the conjugated ciprofloxacin **4** remained unbound in the solution and therefore was detected in the dialysate. However, incubation with 2 µg/mL and 5 µg/mL of trypsin (Figure 4) induced a strong increase of the ciprofloxacin concentration. During the process, PLL is degraded, causing the release of conjugated ciprofloxacin from the nanoparticle. Incubation with higher trypsin concentration led to quicker degradation (Figure 4, black symbols) and confirmed the possibility of controlling the drug release.

### 2.3. Coating of Titanium Samples

**Figure 5:**
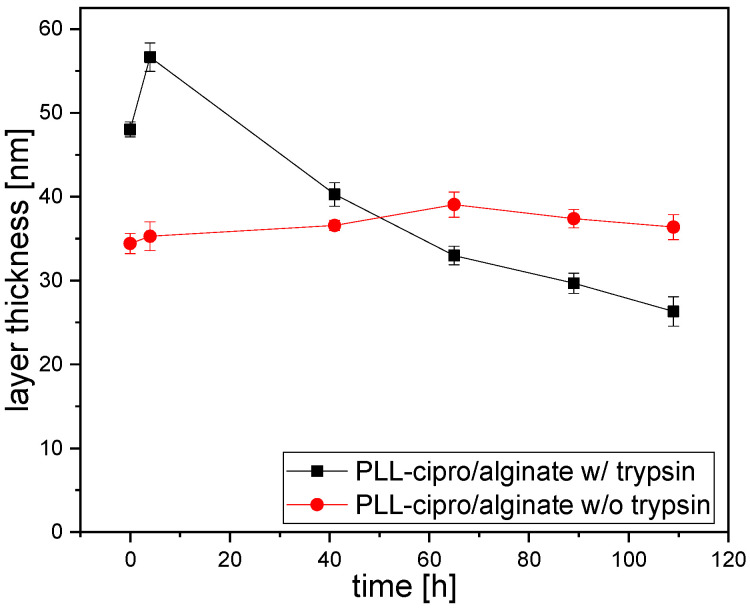
Dry layer thickness of PLL-cipro/alginate coatings as determined by ellipsometry after incubation in PBS buffer with or without addition of trypsin (5 μg/mL) at pH 7.4 and at 37 °C.

The nanoparticle suspensions can be used to coat the surface of titanium implants. The coating process comprises a spray or spin coating, drying, and rising [32,43]. During the coating the nanoparticles form a homogeneous layer [32,43,44]. While chitosan-based nanoparticles directly bind to the negatively charged titanium surface [43], for alginate-based nanoparticles with a negative zeta potential a surface charge reversal is necessary [32]. Here, adsorption of polyethyleneimine was used to obtain a positively charged surface. The thickness of the polyethyleneimine layer was determined by ellipsometry to be around 3.0 ± 1 nm. Subsequently a PLL-ciprofloxacin/alginate (ratio 1:3) nanoparticle dispersion was spray-coated resulting in a dry layer thickness of around 40 ± 6.5 nm. The coating on the titanium was then used for degradation and release experiments. For this purpose, coated titanium plates were incubated in PBS buffer with or without addition of trypsin (5 μg/mL) at pH 7.4 and at 37 °C. In the absence of enzyme the layer thickness does not change, and thicknesses between 35 and 40 nm are determined by ellipsometry for the dried coatings (Figure 5, red curve). For the incubation in buffer with trypsin another coating was prepared, which is slightly thicker at the beginning (~45 nm, Figure 5, black symbols). In this case, the incubation caused an increase in the thickness of the layer in the first few hours (Figure 5, black symbols). This was interpreted to be due the diffusion of trypsin into the layer. Then, over 109 h, a decrease of 45% of the initial layer thickness was observed, clearly indicating the enzymatic degradability of the coatings.

### 2.4. Antimicrobial Efficacy of the Conjugated Ciprofloxacin

**Figure 6:**
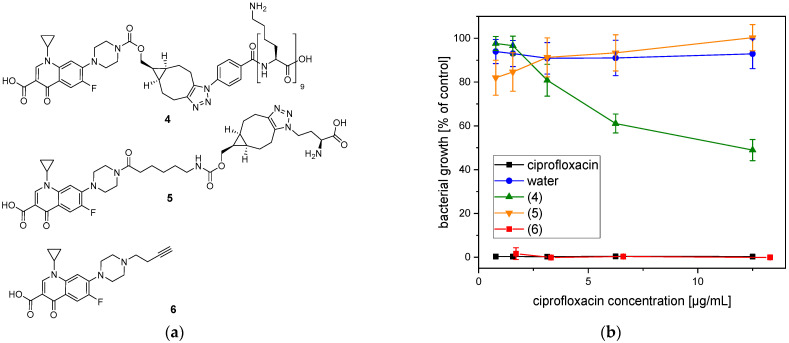
Bacterial growth in the presence of ciprofloxacin modifications. (**a**) Chemical structures of the ciprofloxacin modifications **4**, **5**, and **6**. (**b**) Percentage growth of *S. aureus* (mean ± SD) in the presence of different concentrations of ciprofloxacin modifications (**4**, **5**, **6**), unmodified ciprofloxacin, or water.

Conjugation of ciprofloxacin may affect its antibacterial efficacy. Therefore, the antibacterial effects of the conjugated ciprofloxacin **4** and two other derivatives of ciprofloxacin (**5**, **6**, s. Figure 6a) were investigated against *Staphylococcus aureus*. This bacterial species was used because it is one of the pathogens that often caused orthopedic implant-associated infections [17].

Compound **5** differs from the PLL-conjugated ciprofloxacin **4** in two ways. Cipro floxacin was attached to L-azido homoalanine via 1,3 dipolar cycloaddition, and represents the smallest amino acid that can be linked. By testing its efficacy, the influence of peptide length can be studied. Furthermore, the BCN-moiety in compound **5** is conjugated to ciprofloxacin via a 6-aminohexanoic acid linker in order to reduce any sterical hindrance. In compound **6** the ciprofloxacin is modified with a terminal alkyne moiety by alkylation, much smaller than the groups conjugated to the ciprofloxacin derivatives (**4**, **5**), which also allow conjugation with PLL or other peptides via copper(I)-catalyzed 1,3 dipolar cycloaddition. The reaction between **6** and PLL_9_-N_3_ has been tested, however the copper catalyst could not be fully removed from the product. Indeed, copper ions form very stable complexes with peptides. For instance, Ottaviani et al. studied PLL dendrigraft–Cu(II) complexes, which are of particular interest for their application in bio catalysis [45]. However, the copper is antibacterial and cytotoxic and mediates the generation of reactive oxygen species [46,47], therefore any activity tests with copper-containing samples are not meaningful.

The ciprofloxacin modifications (**4**, **5**, **6**) were added to planktonic *S. aureus* culture at different concentrations. After 24 h, the optical density (600 nm) was measured in order to analyze the bacterial growth. The dilution with MilliQ water (Figure 6, blue symbols) had no influence on the bacterial growth. However, unmodified ciprofloxacin (Figure 5, black symbols) inhibited the growth of *S. aureus* completely independent of the used concentration. The modifications affected the antibacterial efficacy of ciprofloxacin differently. The ciprofloxacin conjugated to PLL (**4**) (Figure 6, green symbols) showed a concentration-dependent antibacterial effect. At a concentration of 12.5 µg/mL, the growth of *S. aureus* was inhibited by 51%. However, the antibacterial efficacy was reduced by decreasing the concentration. In contrast, the ciprofloxacin equipped with butyne group (**6**) (Figure 6, red symbols) inhibited the growth of *S. aureus* completely and the effect was similar to the unmodified ciprofloxacin. In order to clarify, if the antibacterial effect was caused by PLL or bicyclononyne group, the ciprofloxacin modification (**5**) was synthesized. The investigation of the antibacterial effect showed that the substance (**5**) (Figure 6, orange symbols) had no influence on the growth of *S. aureus* and was similar to MilliQ water.

## 3. Discussion

A new nanogel system was prepared by ionotropic gelation of alginate with PLL conjugated with ciprofloxacin. The conjugation of the ciprofloxacin was carried out by modification with a cyclooctyne-group suitable for a copper-free 1,3-cycloaddition with an azide at the peptide sequence. This is a versatile method to link small drug molecules to peptide sequences. The PLL with conjugated ciprofloxacin forms nanoparticles with alginate solutions by ionotropic gelation as evidenced by dynamic light scattering. The nanoparticles are stable in aqueous dispersion, but are degraded upon addition of trypsin. The nanoparticle dispersions were used to prepare stable coatings on titanium by spray coating. The ciprofloxacin is released from the nanoparticles and the coatings only when the PLL sequence is enzymatically cleaved. The results clearly demonstrated the intrinsic capabilities of the systems for application as triggered release systems for antibacterial agents. The effect of the linker on the antibacterial efficacy of the ciprofloxacin used as model antibiotics in this study was also investigated. As a matter of fact, the antibacterial efficacy of the PLL-conjugated ciprofloxacin **4** was reduced. Further derivatives of cipro floxacin were evaluated to elucidate the role of the conjugation chemistry. Compound **5**, also did not show antibacterial activity. This compound did not have a PLL chain, but the same linking group, thus it can be concluded that the acylation of the piperazine ring with a bicyclononatriazole as in the linking group of compounds **4** and **5** hindered the drug from reaching or interacting with the target in the bacteria. On the other hand, compound **6** in which the possible but less sterically demanding linking group is attached via an alkylation at the ciprofloxacin amine shows the same efficacy against *S. aureus* as non-modified ciprofloxacin. Compound **6** shows antibacterial activity, however, needs Cu-ions as catalyst for the click-reaction. Using this compound for preparation of the corresponding nanogels would result in products with compromised biocompatibility.

While acylation of the secondary amino function has been found to be detrimental for activity against a wide range of Gram-negative bacteria [33,48], in which the zwitter-ionic structure of ciprofloxacin is important to pass the outer membrane pore OmpF, acylation is mostly tolerated for activity in Gram-positive bacteria like *S. aureus* having no outer membrane. In this case acylation can even lead to improved antibacterial activities [36]. However, the mechanism of action of ciprofloxacin involves an interaction with gyrase and bacterial DNA. Ciprofloxacin stabilizes the gyrase and bacterial DNA complex, which results in a DNA break [49]. It can be speculated that the linker in **4** and **5** with its structure of three annelated ring systems are too bulky to allow the formation of the complex with gyrase and DNA. The antibacterial activity of the ciprofloxacin conjugated with poly-l-lysin **4** at higher concentrations could be explained by the antibacterial activity of poly-l-lysin itself. PLL shows some antibacterial effect, which however typically needs higher concentrations in particular, when the degree of polymerization is low [50]. In conclusion, despite drug efficiency reduction, the introduced system was able to deliver on-demand molecules without any burst release. Any active substance with amine function can be modified through the same procedure and incorporated into the nanoparticle system.

## 4. Conclusions

Alginate-based nanoscale hydrogel particles were prepared as a drug delivery system that releases the drug triggered by the presence of an enzyme. For this purpose, a generic synthesis route was developed, in which enzymatically cleavable peptides with longer lysine sequences carrying an azide function as the end group were used for ionotropic gelation of the alginate. Via the azide function, active compounds could be attached by copper-free click reaction and then were tightly bound to the nanogel particle. The nanoparticles are stable in suspension, but were degraded by the addition of an enzyme, and the active ingredient was released. The nanoparticle suspension can be also used to coat implant materials like titanium via spray coating. In the deposition process, the nanoparticles are transformed into a homogeneous coating, which is stable unless the enzyme is present. This approach was successfully tested with ciprofloxacin as the antibiotic drug, poly-l-lysine as the model for a peptide, and trypsin as the enzyme. Moreover, the influence of the linker on the activity of the ciprofloxacin was tested. In fact, a significant reduction in the antibiotic activity of the released ciprofloxacin was observed. By studying corresponding derivatives, it was established that the voluminous groups of the linker residue remaining on the ciprofloxacin via an acyl bond reduce the antibiotic activity.

## 5. Materials and Methods

### 5.1. Materials

(1R,8S,9S)-Bicyclo-[6.1.0]-non-4-yn-9-ol, polyethylenimine (PEI), pyridine, alginate, ammonium chloride, trypsin, N-methylmorpholine (NMM), sodium sulfate (Na_2_SO_4_), ciprofloxacin, N,N-dimethylformamide (DMF), 1-hydroxy-1H-benzotriazol (HOBt), trifluoroacetic acid (TFA), N,N-diisopropylethylamine (DiPEA) and diethyl ether were purchased from Sigma-Aldrich (Steinheim, Germany) and used as received. Amino acids, 2-Chlorotrityl chloride resin and N,N′-diisopropylcarbodiimide were purchased from Carbolution Chemicals (St. Ingbert, Germany). Sodium chloride (NaCl), ethyl acetate (EtOAc), and petroleum ether (PE) were purchased from Fisher scientific (Schwerte, Germany). Dichloromethane (CH_2_Cl_2_) and methanol were purchased from VWR (Darmstadt, Germany). All solutions were filtered through 0.22-µm Millex-GP (polyethersulfon; Sigma-Aldrich) filters before use. Titanium alloy substrates were cut into 1 × 1 cm^2^ squares. Dichloromethane, PE, DMF, diethyl ether were purified with a Solvent Purification System SPS-800 from MBraun (München, Germany) before use.

### 5.2. Synthesis of BCN-O(CO)O(4-NO2-Ph) ***1***

First, 100 mg (0.666 mmol) (1R,8S,9s)-bicyclo6.1.0non-4-yn-9-yl-methanol (BCN-OH) were dissolved in 17 mL dry CH_2_Cl_2_. About 134 μL (1.664 mmol) of pyridine and 168 mg (0.831 mmol) of 4-nitrophenyl chloroformiate were added to the solution. The mixture was stirred for 20 min at 22 °C before being quenched by the addition of 20 mL of saturated ammonium chloride solution. The mixture was extracted with CH_2_Cl_2_ (3 × 20 mL). The combined organic layers were dried over Na_2_SO_4_ and concentrated under reduced pressure. Flash chromatography through silica gel was performed to purify the residue. A mixture of light petroleum-EtOAc (95–5) and (90–10) was used for the chromatography.

A white solid was obtained: 180 mg. Yield = 85%

^1^H-NMR (300 MHz, CDCl_3_) δ [ppm] = 8.26–8.31 (m, 2H); 7.37–7.42 (m, 2H); 4.4 (d, 2H); 2.38–2.26 (m, 4H); 2.25–2.21 (m, 2H); 1.66–1.57 (m, 2H); 1.49 (p, 1H); 1.11–1.02 (m, 2H). (see Figure S1)

### 5.3. Synthesis of BCN-O(CO)HN-Ciprofloxacin ***2***

At 82 °C, 42 mg (0.127 mmol) of ciprofloxacin was dissolved in 7 mL of dry DMF. After the complete dissolution, 38 mg (0.38 mmol) of *N*-methylmorpholine (NMM) and a solution of 40 mg (0.127 mmol) BCN-O(CO)O(4-NO_2_-Ph) in 2 mL DMF were added. The solution was stirred for 24 h at 23 °C. Then, DMF was evaporated under reduced pressure. It is important to stop the evaporation before the end to keep 0.5 mL of the solution. The residue was purified by flash chromatography through silica gel. A mixture of CH_2_Cl_2_/MeOH (95–5 and 90–10) was used. After vacuum drying, a yellow solid was obtained: 45 mg. Yield = 70%

^1^H-NMR (300 MHz, CDCl_3_) δ [ppm] = 4.17 (d, J = 8.2 Hz, 2H), 3.70–3.63 (m, 4H); 3.48 (tt, J = 7.1, 4.0 Hz, 1H); 3.29–3.21 (m, 4H); 2.32–2.10 (m, 6H); 1.62–1.46 (m, 2H); 1.37–1.29 (m, 3H); 1.17–1.10 (m, 2H); 0.96–0.86 (m, 2H). (see Figure S2)

MS-ESI (m/z): found 508.22 [M + H]^+^; 530.20 [M + Na]^+^; 1037.42 [2 M + Na]^+^ (calculated for C_28_H_30_FN_3_O_5_= 507.22)

### 5.4. Synthesis of Poly-l-lysine-Ciprofloxacin (***4***)

PLL_9_-N_3_ **3** was synthesized using automated standard solid phase-supported peptide (SPPS) synthesis utilizing a Syro II Peptide synthesizer from MultiSynTech. The first Fmoc-protected Lys was loaded onto a 2-chlorotrityl resin (capacity 0.48 mmol/g) in the presence of Et_3_N in dichloromethane at 23 °C for over 12 h. The loading of the resin was determined after Fmoc-cleavage in the presence of a solution of 20 w% piperidine in DMF analyzing the concentration of the fulvene-piperidine adduct at 309 nm by UV-Vis spectrometry. Coupling of further Fmoc-protected lysines and 4-azidobenzoic acid was achieved after Fmoc-cleavage (Piperidine 20 w% in DMF) using the acid as well as DIC/HOBt/DiPEA:1/1/1 as the coupling mixture in four-fold excess. Between all cleavage and coupling steps the resin was washed with DMF. Final cleavage of the peptide from the resin was achieved in the presence of TFA at 23 °C over a period of 5 min and PLL_9_-N_3_ **3** was obtained from the cleavage solution by precipitation with cold diethylether.

For this synthesis of **4**, 40 mg of PLL_9_-N_3_ **3** were added to a solution of 18 mg BCN-O(CO)HN-Ciprofloxacin (0.035 mmol) in 4 mL. The mixture was stirred overnight at 23 °C before the product was precipitated with diethyl ether, re-dissolved in DMF, and re-precipitated. Finally, the solid was washed with diethyl ether until a white powder was obtained. Yield = 81%

^1^H-NMR (300 MHz, D_2_O) δ [ppm] relevant peaks: 8.55 (s, 1H, CIP); 7.89 (m, 2H, PLL); 7.13–7.49 (m, 4H, CIP/PLL); 2.99 (m, 24H, PLL); 1.98–1.29 (PLL) (see Figure S3). A comparison of the typical absorbance spectra of **4** and of the commercial ciprofloxacin can be found in Figure S10.

### 5.5. Synthesis of ***5***

Synthesis of molecule **5** is performed in three steps.

Step 1:

Ciprofloxacin 165.5 mg (0.5 mmol) and N,N-diisopropylethylamine 0.5 mL (2.9 mmol) were mixed in 3 mL of dry CH_2_Cl_2_ and 185 μL trimethylsilyl chloride. The solution turned yellow. Separately, 6-((tert butoxycarbonyl)amino)hexanoic acid 173 mg (0.75 mmol), PyAOP 417 mg (0.8 mmol), and DIPEA 350 μL (2 mmol) were dissolved in 2 mL of dry CH_2_Cl_2_. The two solutions were combined and stirred overnight at 23 °C. 60 mL H_2_O were added and the aqueous solution was extracted with 3 × 80 mL CH_2_Cl_2_. The combined organic layers were washed with 60 mL H_2_O, 10% *w*/*w* citric acid in water, saturated aqueous NaHCO_3_ solution, brine, and 2 × 50 mL H_2_O. The organic phase was dried over Na_2_SO_4_ and concentrated under reduced pressure.

A yellow solid was obtained: 190 mg. Yield = 70%.

^1^H-NMR (CDCl3, 300 MHz), δ 1.18–1.23 (2H, m), 1.36–1.56 (15H, m), 1.63–1.74 (2H, m), 2.39 (t, J = 7.5 Hz, 2H), 3.12 (dt, J = 6.7 Hz, 2H). 3.25–3.39 (4H, m), 3.51–3.59 (1H, m), 3.67–3.90 (4H, m), 4.56 (1H, bs), 7.36 (d, J = 7.1 Hz, 1H), 7.99 (d, J = 12.9 Hz, 1H), 8.72 (s, 1H), 14.9 (1H, bs). MS-ESI (m/z): calculated 545.28; found 567.82 (z = 1, [M + Na]^+^); 1111.53 (z = 1/2, [M + Na]^+^), (z = 1/3, [M + Na]^+^).

The trifluoroacetic acid (TFA) salt was obtained by stirring it overnight, in 16.7% TFA/CH_2_Cl_2_, at room temperature and by removing the solvent with methanol as co-solvent. The resulting solid was re-precipitated with diethyl ether. A yellow pale solid was obtained: 110 mg. Yield = 90%

^1^H-NMR (CD3OD, 300 MHz), δ 1.22 (m, 2H) 1.54–1.37 (m, 4H), 1.63–1.77 (m, 4H), 2.52 (t, J = 7.3 Hz, 2H), 3.05–2.86 (m, 2H), 3.46–3.33 (m, 4H), 3.87–3.70 (m, 5H), 7.55 (d, J = 7.2 Hz, 1H), 7.81 (d, J = 13.2 Hz, 1H), 8.72 (s, 1H). 19F NMR (CDCl3, 282 MHz), δ −75.36, −121.56.

Step 2:

Total of 100 mg (0.127 mmol) of the substance synthesized in step 2 was dissolved in 2.65 mL dry DMF. After complete dissolution, 78 μL (mmol) NMM and a solution of 56.5 mg (mmol) BCN-O(CO)O(4-NO_2_-Ph) in 2 mL DMF were added and stirred for 24 h at 23 °C. DMF was evaporated under reduced pressure (it is important to stop the evaporation before the end in order to keep 0.5 mL of the solution, otherwise the isolated yield drops significantly). The residue was purified by flash chromatography through silica gel. Solvent: CH_2_Cl_2_/MeOH (95–5 and 90–10). A yellow solid was obtained: 50 mg. Yield = 45%.

^1^H-NMR (DMF, 600 MHz), δ 8.75 (s, 1H), 7.97 (d, J = 13.2 Hz, 1H), 7.73 (d, J = 7.4 Hz, 1H), 7.00 (t, J = 5.8 Hz, 1H), 4.09 (d, J = 8.1 Hz, 2H), 3.94 (tt, J = 7.3, 4.1 Hz, 1H), 3.79 (m, 4H), 3.42 (m, 4H), 3.11 (td, J = 7.1, 5.8 Hz, 2H), 2.45 (t, J = 7.5 Hz, 2H), 2.30–2.14 (m, 6H), 1.66–1.22 (m, 15H), 0.95–0.85 (m, 2H).

Step 3:

About 0.024 mmol of L-azido homoalanine were added to a solution of 15 mg of the substance synthesized in step 3 (0.024 mmol) in 1 mL DMF. The solution turned from yellow to transparent. The mixture was stirred over night at 23 °C, before the product was precipitated with diethyl ether, re-dissolved in DMF, and re-precipitated. Finally, the solid was washed with diethyl ether and a white-yellow solid (80–90%) was obtained. A white solid was obtained (5): 17 mg. Yield: 87%

^1^H-NMR (DMF, 600 MHz), δ 8.75 (s, 1H), 7.97 (d, J = 13.2 Hz, 1H), 7.74 (d, J = 7.5 Hz, 1H), 7.05 (t, J = 5.8 Hz, 1H), 4.71 (m, 1H), 4.33 (t, J = 6.3 Hz, 1H), 4.12 (m, 2H), 3.95 (tt, J = 7.3, 4.0 Hz, 1H), 3.83–3.75 (m, 4H), 3.48–3.36 (m, 4H), 3.14–3.08 (m, 2H), 3.08–3.00 (m, 2H), 2.88–2.81 (m, 2H), 2.60 (m, 2H), 2.45 (t, J = 7.5 Hz, 2H), 2.23–2.07 (m, 2H), 1.68–1.31 (m, 14H), 1.16 (m, 1H), 1.00 (m, 1H). (see Figure S5). A comparison of the typical absorbance spectra of **5** and of the commercial ciprofloxacin can be found in Figure S10.

### 5.6. Synthesis of ***6***

NaHCO_3_ (0.187 g, 0.0022 mol) and ciprofloxacin (0.5 g, 0.0015 mol) were dissolved in 150 mL of dry DMF at 80 °C. Once a clear solution is obtained, the reaction mixture was degassed with N_2_ for 15 min. Finally, 4-bromo-1-butyne (1 g, 0.0075 mol) was added and the solution was stirred for 24 h at 21 °C. After 24 h, the DMF was removed under reduced pressure by adding toluene as a co-solvent. The resulting solid was purified by flash chromatography on silica gel. A mixture of CH_2_Cl_2_ and MeOH (97–3) was used for the chromatography. A white solid was obtained: 172 mg. Yield = 29%

^1^H-NMR (700 MHz, CDCl_3_) δ [ppm] = 8.70 (s, 1H); 7.95 (d, *J* = 13.1 Hz, 1H); 7.29 (d, *J* = 7.2 Hz, 1H); 3.47 (tt, *J* = 7.1, 3.9 Hz, 1H); 3.35–3.25 (m, 4H); 2.65 (m, 6H); 2.39 (m, 2H); 1.95 (t, *J* = 2.6 Hz, 1H); 1.36–1.27 (m, 2H); 1.13 (m, 2H). (see Figure S4)

### 5.7. Purification of Alginate

Alginate was purified according to the method outlined by Tolle et al. [32]. About 2 g of commercial alginate were dissolved in 30 mL of deionized H_2_O. This solution was dialyzed over three days against deionized H_2_O with three medium changes a day (14 kDa MW cutoff). The solution was stirred overnight with 0.5 g activated carbon per gram of alginate. After being stirred for 15 h, the solution was filtered for removal of the activated carbon. Finally, the filtrate was freeze-dried and stored at −20 °C to avoid the water uptake of the hygroscopic material.

^1^H NMR (D_2_O, 600 MHz): 3.96 ppm (m)

FT-IR, ATR, ν [cm^−1^]): 3325, 1596, 1407, 1297, 1088, 1028, 625.

### 5.8. Preparation of Alginate/α-PLL-BCN-O(CO)HN-Ciprofloxacin Nanoparticles

Solutions of 1 mg/mL alginate (solution 1) and PLL-ciprofloxacin (solution 2) were prepared in filtered pure water. The nanoparticles were prepared by mixing solutions 1 and 2 at different ratios. The tested ratios were 1:4, 1:3, 1:2, and 1:1 (Alg:PLL-ciprofloxacin). Then, particle size, stability measurements, and degradation study were carried out using a Zetasizer Nano ZS from Malvern Instruments (Malvern, UK). Disposable sizing cuvettes (DTS0012) were used for size measurements. Malvern Zetasizer Software Version 7.03 was used for data evaluation.

### 5.9. Coating of Alginate/PLL-Ciprofloxacin Nanoparticles on Ti Plates

Before being coated with alginate nanoparticles, the substrates were polished, and cleaned through ultra-sonification in water, dichloromethane, acetone, methanol, and MilliQ water. The titanium substrates were first coated with a polyethylenimine (PEI) layer. The substrate was immersed for one minute in 5% (*w*/*w*) PEI solution, washed with pure water, and dried under nitrogen. Then, the substrates were spray-coated (three minutes) with an airbrush Aztek A470 from Testors (Vernon Hills, IL, USA), depositing approximately 20 µL of the Alg/PLL-ciprofloxacin nanoparticle solution. The particle formation process was carried out in MilliQ water with both 1 mg/mL of sodium alginate and peptides. Subsequently, the Ti plates were rinsed with H_2_O in an ultrasonic bath for 15 min. After the plates were dried under nitrogen flow, ellipsometry was used to measure the coating thickness. Layer thicknesses were determined using a Multiskop from Optrel (Sinzing, Germany) in the ellipsometry mode. Uncoated titanium plates were used as reference. Data were collected in the x, y-mode at 70° as mean value of 16 data points in total. Evaluation of the data was carried out using Elli Version 3.2 from Optrel.

### 5.10. Stability of Nanoparticles in Aqueous Dispersion

A solution of alginate/PLL-ciprofloxacin nanoparticle was prepared with a ratio of 1:3 in phosphate-buffered saline solution pH = 7.4 and added to the disposable sizing cuvettes. The particle’s behavior was analyzed via dynamic light scattering with a Zeta Nano ZS device at 37 °C for 50 h.

### 5.11. Stability of Coatings on Titanium

The coating stability was tested by immersing the coated substrate in buffer solution (PBS, pH = 7.4) at 37 °C for 109 h. The test was performed in triplicate to ensure the reproducibility. Before each thickness measurement via ellipsometry, the samples were washed with H_2_O and dried under nitrogen flow.

### 5.12. Enzymatic Degradation of Nanoparticles and Coatings

The degradation study was carried out by incubation in trypsin-containing buffer solution (PBS, pH = 7.4). A PLL hydrolysis reaction was initiated by adding trypsin to obtain a final concentration of 5 μg/mL at 37 °C. The solutions were renewed daily to ensure the degradation capability of trypsin. Before each measurement, the samples were washed with H_2_O and dried under nitrogen flow. The test was performed in triplicate to ensure reproducibility.

The degradation of the Alg/peptide nanoparticles was accomplished through the addition of trypsin to the nanoparticle dispersion. Briefly, 1 mL Alg/PLL-ciprofloxacin nanoparticle suspensions were filled in a sizing cell and maintained at 37 °C. Trypsin solution was added in order to obtain final concentrations of 2.5 µg/mL. The degradation process was monitored via consecutive size measurements using the Zetasizer Nano ZS.

### 5.13. Enzyme-Triggered Release

For the tests 3 × 6 mL of particle solutions were prepared. Two solutions were treated with a concentration of 2 μg/mL and 5 μg/mL of trypsin. The last was not treated with enzyme and was used as a control. After trypsin was added, the solutions were incubated at 37 °C. Then, every 15 min, 1 mL of each sample was collected and inserted into a Vivaspin tube containing two vertical membranes with a cutoff of 5 kDa. The tube was centrifuged, and the ciprofloxacin concentration was determined using a V-630 UV-VIS Spectrophotometer from JASCO and a quartz glass Ultra-Micro Cell. A calibration curve was prepared by plotting different concentrations of commercial ciprofloxacin hydrochloride versus their absorbances at λ = 278 nm and found to be linear in the concentration range of 2 to 10 µg/mL. This curve was then used to determine the concentration of ciprofloxacin released during the degradation process.

### 5.14. Antibacterial Efficacy of the Conjugated Ciprofloxacins

The antimicrobial efficacy of the conjugated ciprofloxacins **4**, **5**, and **6** were investigated against *Staphylococcus aureus* (DSM 799, German Collection of Microorganisms and Cell Cultures, Braunschweig, Germany). *S. aureus* was cultured in tryptone soya broth supplemented with 10% yeast extract (TSBY) for 18 h at 37 °C. The preculture was adjusted to an optical density (600 nm) of 0.001 in TSBY and added to a 96-well plate (100 µL/well). The conjugated ciprofloxacins were diluted 1:2 in TSBY as a five-fold dilution series. In order to prevent nutrient reduction, the first dilution was prepared in two-fold TSBY. In the same way, ciprofloxacin and MilliQ water were diluted and served as controls. The dilution series were added to *S. aureus* in a 96-well plate (100 µL/well) and cultivated for 24 h under shaking (400 rpm) at 37 °C. Afterwards, the optical density (600 nm) was measured by microplate reader (infinite M200PRO, Tecan, Männedorf, Switzerland). The absorption values were normalized against the positive control, which was a *S. aureus* culture in TSBY under the same cultivation conditions.

### 5.15. Spectroscopic Characterization

A FTIR Equinox 55 instrument from Bruker (Billerica, MA, USA) equipped with an attenuated total reflection accessory with a zinc selenide crystal (Harrick Scientific Products, Pleasantville, NY, United States) and a mercury cadmium telluride detector was used to perform Fourier transform infrared spectroscopy. The following condition were used for FTIR measurements: wavelength range between 4000 cm^−1^ and 550 cm^−1^, 10 KHz and 32 scans per sample. The evaluation was carried out with the software OPUS from Bruker (version 4.0.24).

^1^H-NMR measurements were performed with AV III HD 300N and AV 600 instruments (Bruker, Billerica, MA, USA), with magnetic field strength of 300 and 600 MHz. The different deuterated solvents were purchased from Deutero. The evaluation was carried out with the analytical software MestReNova from MESTRELAB.

Electrospray ionization mass spectrometry (ESI-MS) was conducted on a LTQ-Orbitrap Velos spectrometer (Thermo Fisher Scientific, Waltham, MA, USA). A voltage of 2.3 to 2.8 kV (positive) or 1.7 to 2.5 kV (negative) was applied. The sample was dissolved in methanol (c = 50 µg/mL) and 0.1 mg/mL trimethyltetradecylammonium bromide was added. The flow rate was adjusted to 0.1 µL/min.

## Supplementary Materials

The following are available online at https://www.mdpi.com/article/10.3390/antibiotics10060653/s1. Figure S1: ^1^H-NMR spectrum and peak assignment for **1** in deuterated CDCl_3_ with peak assignment. Figure S2: ^1^H-NMR spectrum and peak assignment for BCN-O(CO)HN-ciprofloxacin in deuterated CDCl_3_. Figure S3: ^1^H-NMR spectrum and peak assignment for ciprofloxacin-poly-l-lysine in deuterated H_2_O. Figure S4: ^1^H-NMR spectrum and peak assignment for ciprofloxacin **6** in deuterated CDCl_3_. Figure S5: ^1^H-NMR spectrum and peak assignment for substance **5** in deuterated DMF. Figure S6: 2D-COSY spectrum of substance **5** in deuterated DMF. Figure S7: NH-HMBC spectrum of substance **5** in deuterated DMF. Figure S8: 2D-HMBC spectrum of substance **5** in deuterated DMF. Figure S9: FT-IR spectra of PLL_9_-N_3_ (**3**) and ciprofloxacin-poly-l-lysine (**4**). Figure S10: UV-Vis absorbance spectra of ciprofloxacin-L-lysine, substance **5**, substance **6**, and commercial ciprofloxacin.
