# Supplementary material for: Enzyme-Responsive Nanoparticles and Coatings Made from Alginate/Peptide Ciprofloxacin Conjugates as Drug Release System
Source: Antibiotics (Basel). 2021 May 29;10(6):653. doi: 10.3390/antibiotics10060653 (PMC8226786; doi:10.3390/antibiotics10060653)
Supplement: Supplementary file 1 [file antibiotics-10-00653-s001.zip › antibiotics-1205732-supplementary.pdf]

Supporting information

Enzyme-Responsive Nanoparticles and Coatings Made from Alginate/Peptide Ciprofloxacin Conjugates as Drug Release System

Y. Bourgat[1], C. Mikolai [2,3], Meike Stiesch[2,3] P. Klahn [4], and H. Menzel[1]

1) Technische Universität Braunschweig, Institute for Technical Chemistry, Hagenring 30, 38106 Braunschweig, Germany

2) Department of Prosthetic Dentistry and Biomedical Materials Science, Hannover Medical School, Carl-Neuberg-Straße 1, 30625 Hanover, Germany

3) Lower Saxony Centre for Biomedical Engineering, Implant Research and Development (NIFE), Stadtfelddamm 34, 30625 Hanover, Germany

4) Technische Universität Braunschweig, Institute of Organic Chemistry, Hagenring 30, 38106 Braunschweig , Germany

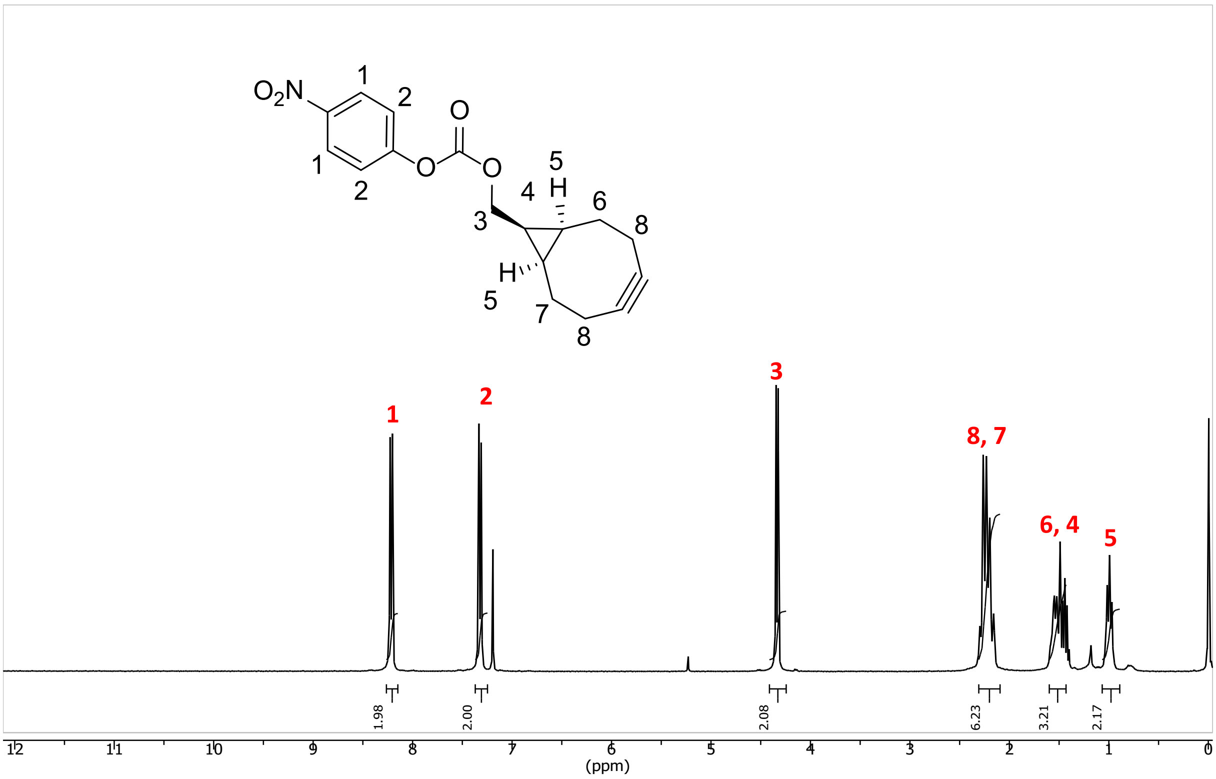

Figure S1: ^{1}H-NMR spectrum and peak assigment for BCN-O(CO)O(4-NO$_2$-Ph) ***1*** *in deuterated CDCl$_3$*

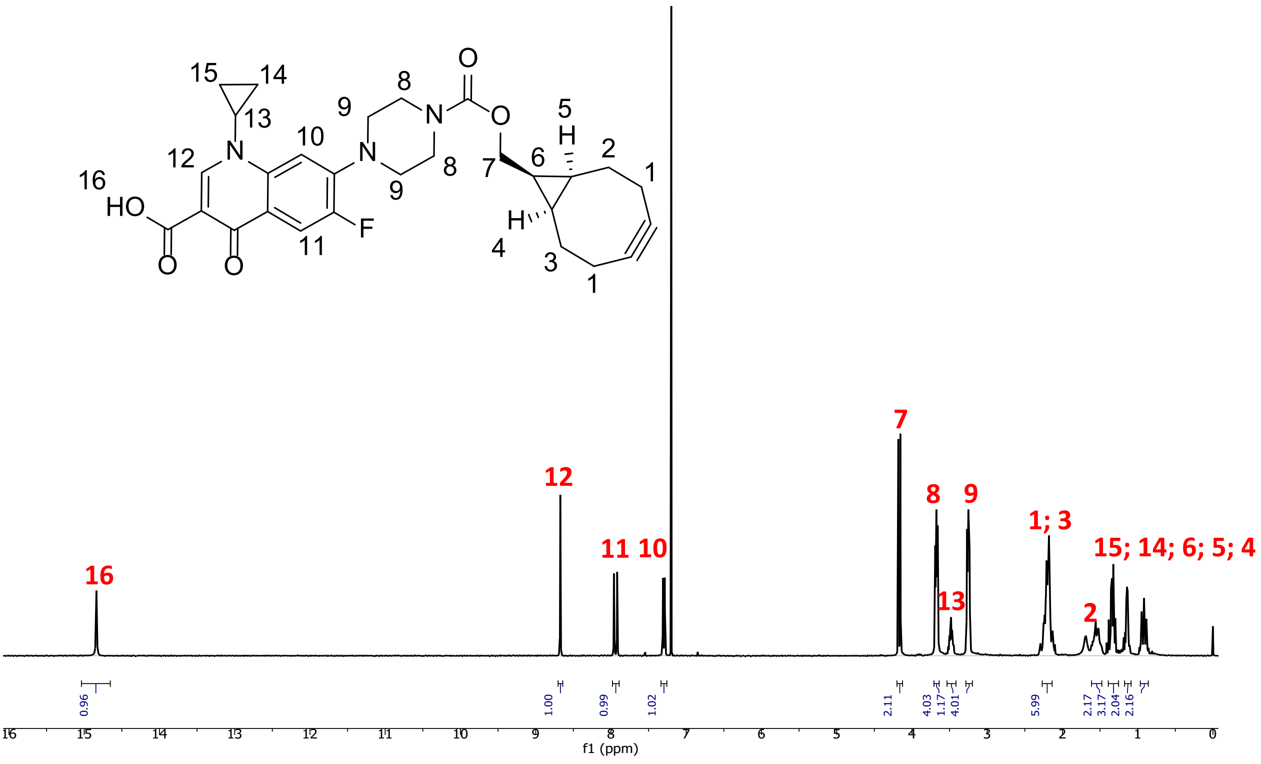

Figure S2: ^{1}H-NMR spectrum and peak assignment for BCN-O(CO)HN-Ciprofloxacin ***2*** *in deuterated CDCl$_3$.*

Signals at 8.67, 7.92, and 7.29 ppm were found, which represent the quinolone framework protons of ciprofloxacin. The presence of a doublet at 4.17 ppm confirmed the presence of the BCN moiety. Moreover, four protons of the nitrophenyl leaving group, disappeared.

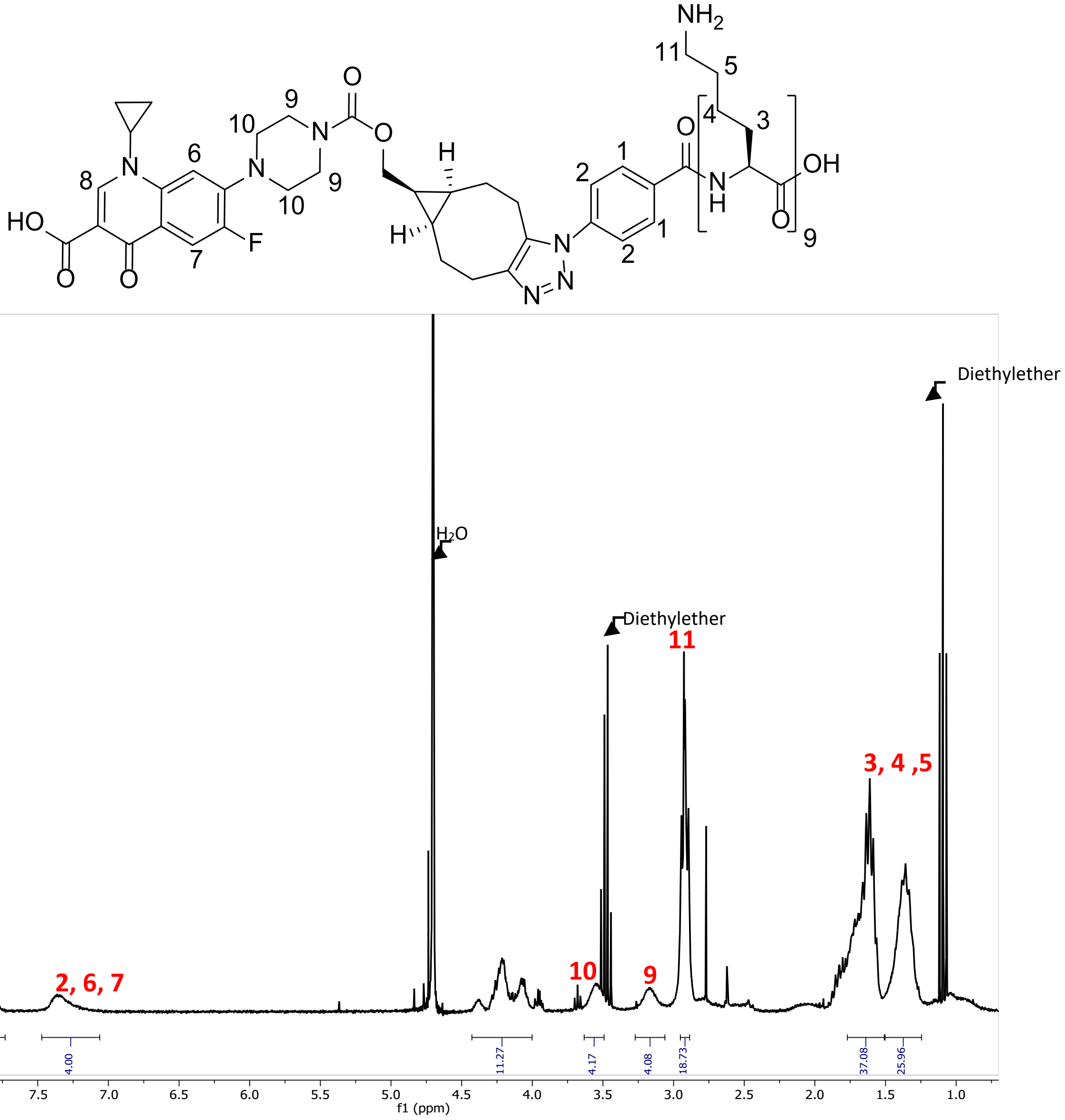

*Figure S3: ^{1}H-NMR spectrum and peak assignment for Ciprofloxacin-poly-L-lysine **4** in deuterated H_2O.*

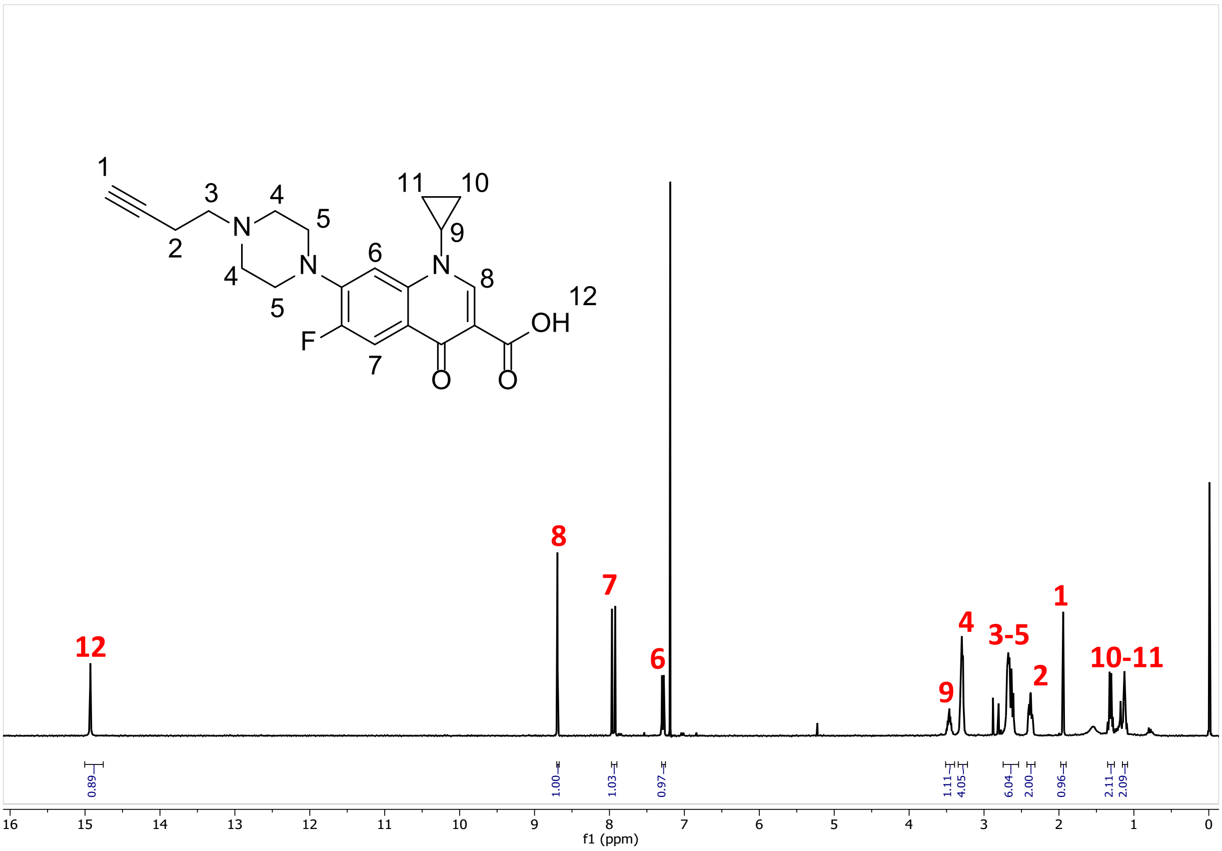

*Figure S4: ^{1}H-NMR spectrum of propargyl modified Ciprofloxacin **6** in deuterated $CDCl_3$*

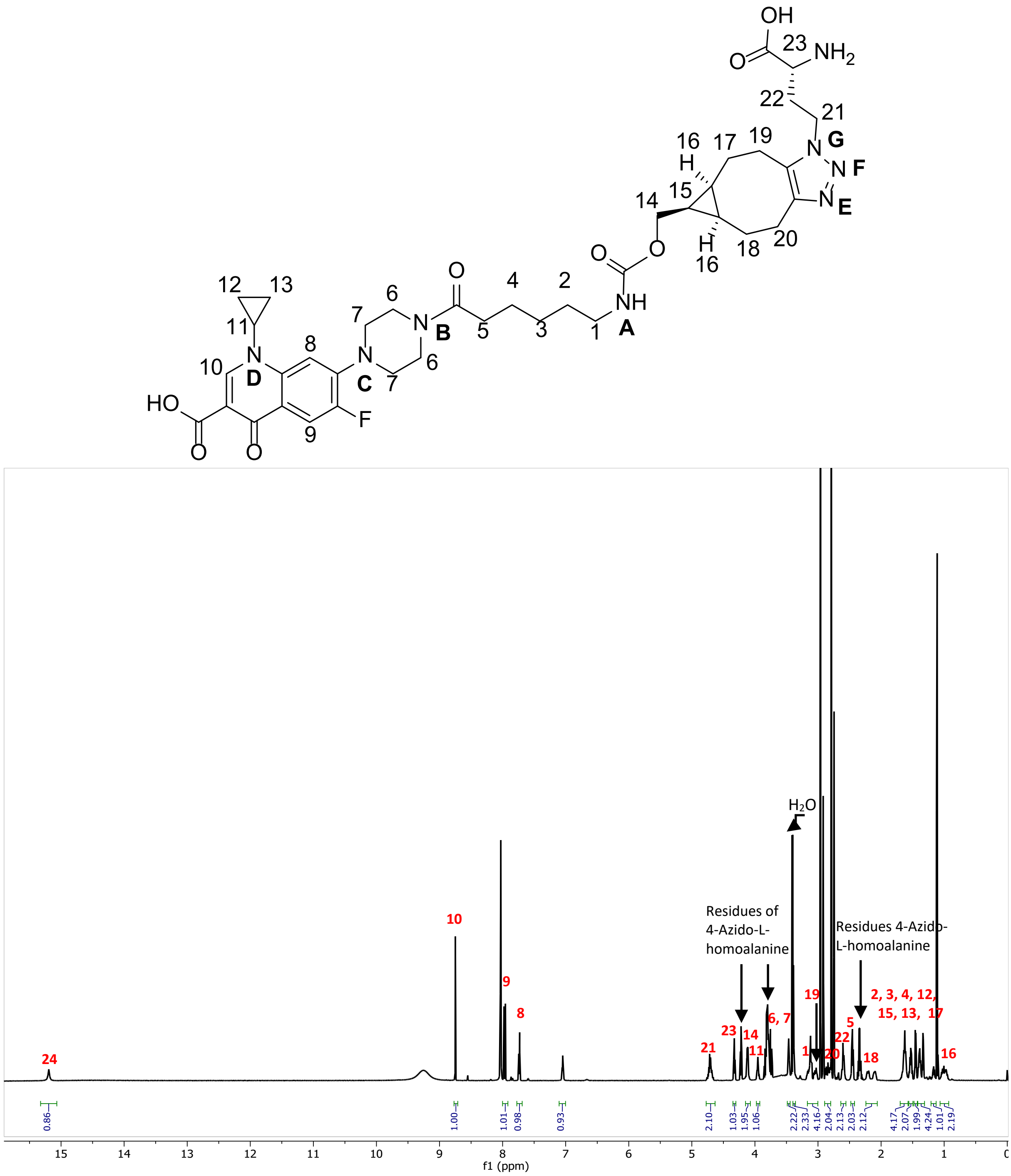

*Figure S5: ^{1}H-NMR spectrum of substance **5** in deuterated DMF.*

The spectrum shows some remains of the reagent 4-azido-homoalanine, which could not be removed completely by precipitation of the polymer. With the help of 2D- and NH-HMBC spectra the coupling of the ciprofloxacin was established. For 4-azido-homoalanine no antibacterial effect was observed in preliminary tests, therefore, **5** was used for the experiments.
NH-HMBC and 2D-COSY (s. Figure S 7 and Figure S 6) spectrum of **5** and 4-azido-homoalanine were performed and used to highlight 4-azido-homoalanine as residue.
^{14}N-NMR (DMF, 600 MHz): -26.63 (F), -132.59 (4-azido-homoalanine) -135.18 (G), -230.03 (D), -298.01 (A), -310.22 (4-azido-homoalanine), -314.38 (C).

Morevoer, 2D-HMBC (s. Figure S8) analysis highlighted the disappearance of the triple bond signal at 100.25 ppm and the appearance of C=C double bond signals of 1,2,3-triazoles bond at 135.34 and 144.93 ppm.

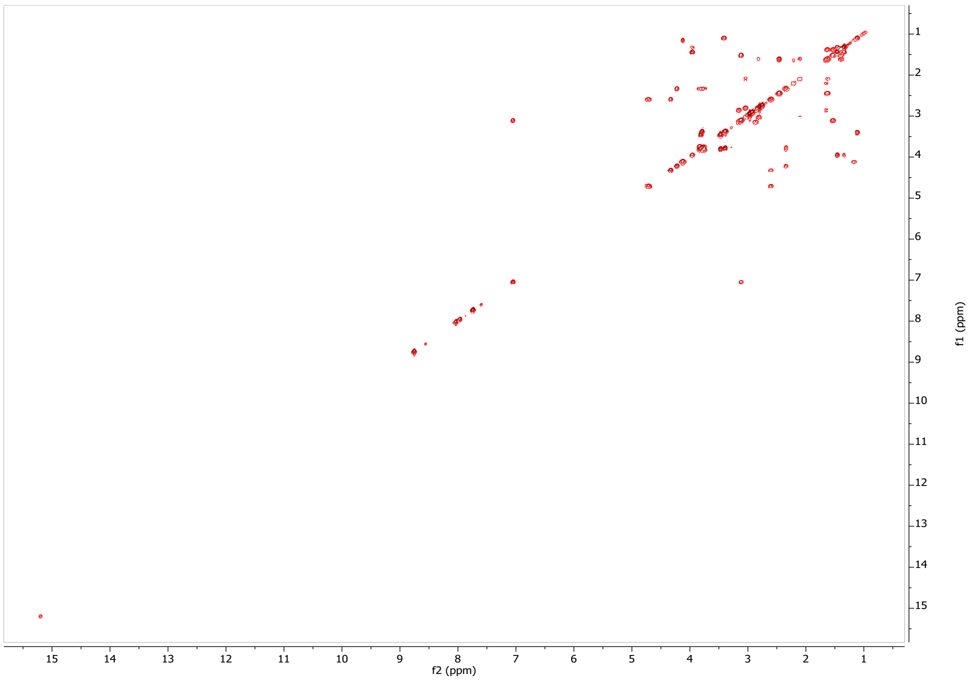

*Figure S6: 2D-COSY spectrum of substance **5** in deuterated DMF*

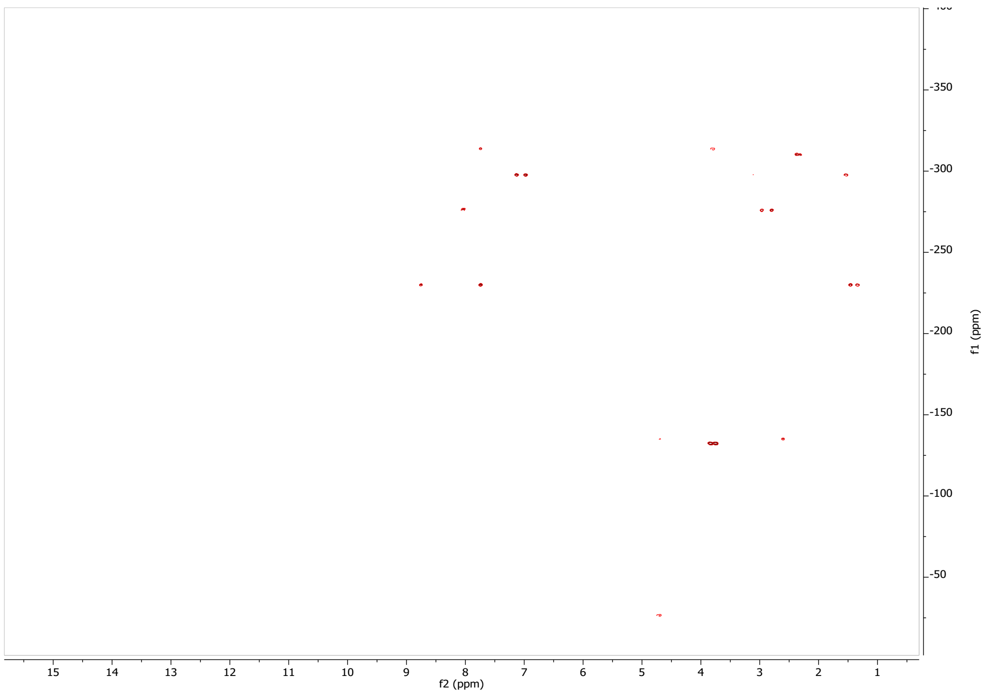

*Figure S7:NH-HMBC spectrum of substance **5** in deuterated DMF*

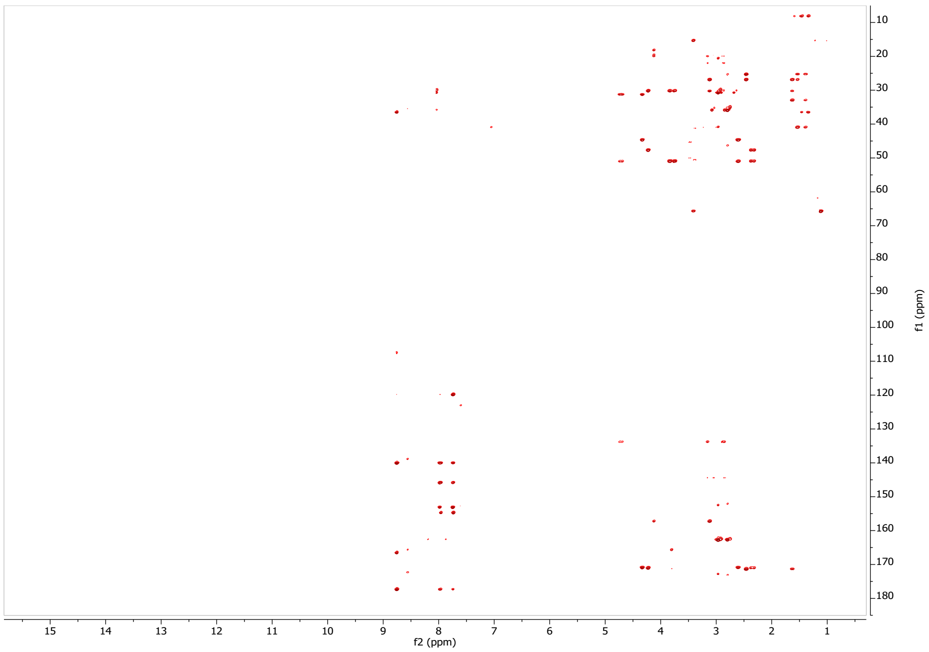

*Figure S8: 2D-HMBC spectrum of substance **5** in deuterated DMF*

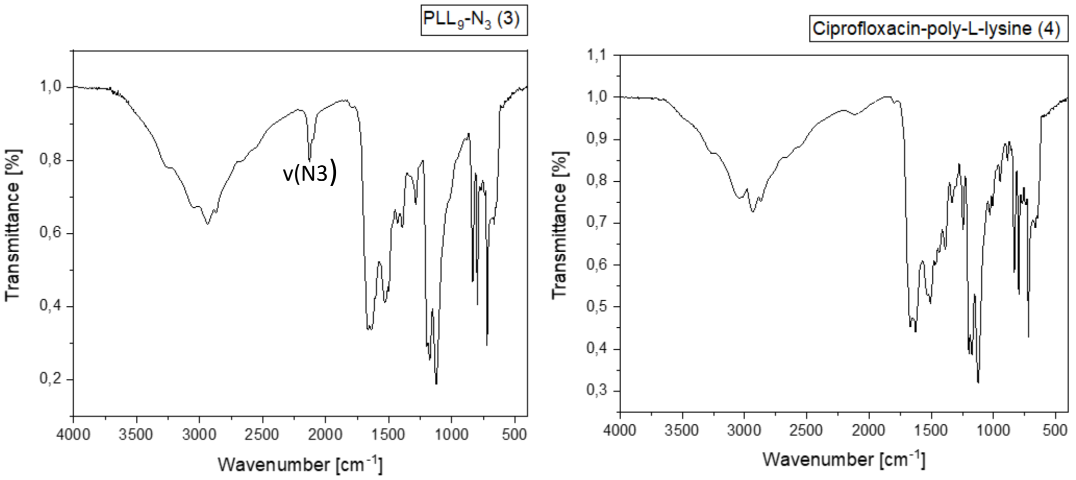

*Figure S9: FT-IR spectra of PLL9-N3 (**3**) and Ciprofloxacin-poly-L-lysine (**4**)*

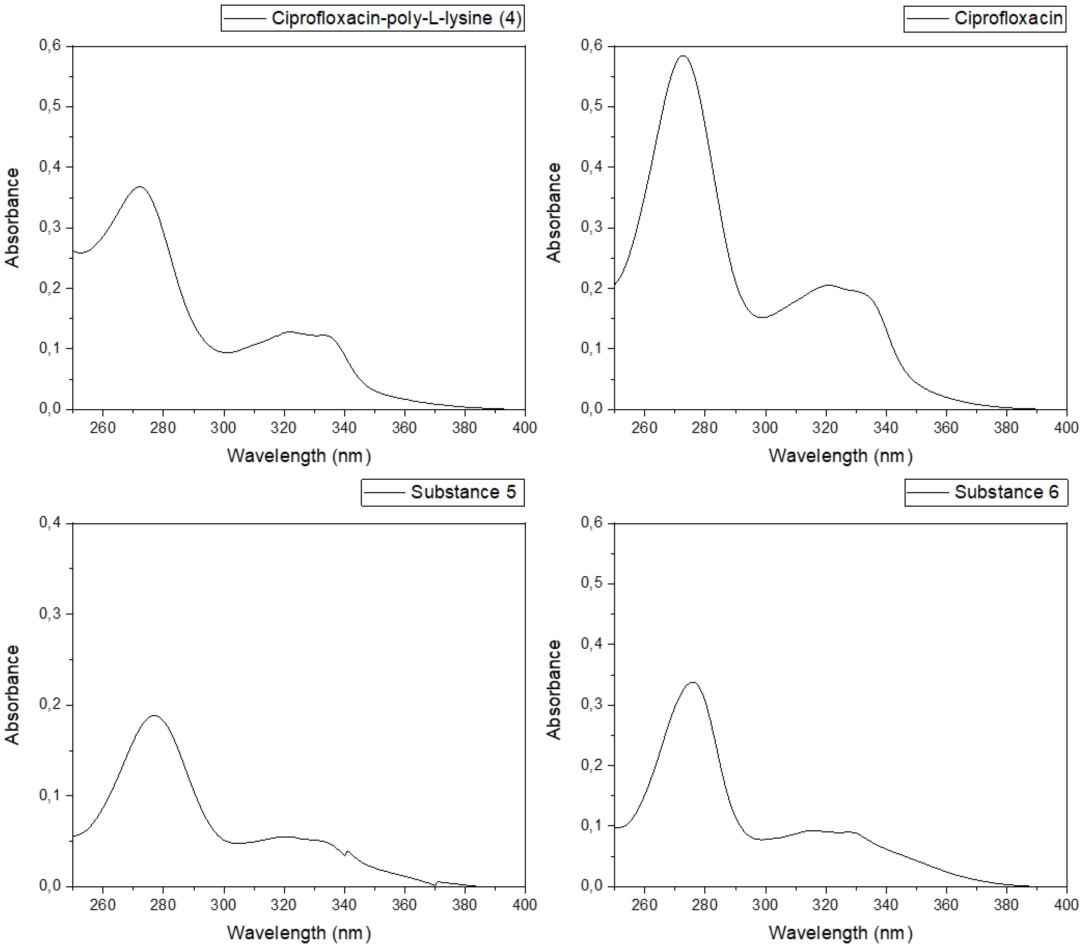

*Figure S10: UV-Vis absorbance spectra of Ciprofloxacin-L-lysine, substance **5**, substance **6** and commercial Ciprofloxacin*
